# Case Report: Overlap syndrome with concurrent polymyositis, masticatory myositis, and lymphocytic thyroiditis in a dog

**DOI:** 10.3389/fvets.2026.1807901

**Published:** 2026-06-04

**Authors:** Matthew I. Crawford-Jennings, Ryan C. Moreno, G. Diane Shelton, Kayla M. Fowler, Christina R. Vezza, Richard L. Shinn, Rell L. Parker, Ashley R. Wilkinson

**Affiliations:** 1 Department of Clinical Sciences, North Carolina State University College of Veterinary Medicine, Raleigh, NC, United States; 2 Department of Small Animal Clinical Sciences, Virginia-Maryland College of Veterinary Medicine, Virginia Polytechnic Institute and State University, Blacksburg, VA, United States; 3 Comparative Neuromuscular Laboratory, University of California, San Diego, La Jolla, CA, United States; 4 Pinnacle Veterinary Specialists, Glen Mills, PA, United States; 5 Veterinary Medical Center of Long Island, West Islip, NY, United States

**Keywords:** canine, creatine kinase, electrodiagnostics, immune-mediated, inflammatory myopathy, lymphocytic hypothyroidism

## Abstract

**Case presentation:**

A 4-year-old spayed female mixed breed dog presented for chronic upper airway noises and dysphagia. The dog presented for inspiratory stridor and expiratory stertor. Serum creatine kinase activity was 3,296 IU/L, and cholesterol and triglyceride levels were elevated at 353 mg/dL and 201 mg/dL, respectively. In-house thyroxine levels were below the reference range, prompting the submission of a comprehensive thyroid profile to an outside reference laboratory. Head and cervical CT with contrast showed perivertebral/perilaryngeal muscle thickening and contrast enhancement. Electrodiagnostics of the pelvic limbs and head showed diffuse spontaneous activity with normal motor nerve conduction velocity. Muscle biopsies revealed an inflammatory myopathy in both masticatory muscles and pelvic limb muscles. The serum 2M antibody titer was positive. Comprehensive infectious PCR and serology testing were negative. The constellation of findings is compatible with an overlap syndrome of masticatory myositis and immune-mediated polymyositis. A comprehensive thyroid panel consisting of total thyroxine, total triiodothyronine, free thyroxine by dialysis, T4 and T3 autoantibodies, thyroid-stimulating hormone, and thyroglobulin autoantibodies later returned supported results for lymphocytic thyroiditis.

**Intervention and outcome:**

Treatment with immunosuppressive doses of prednisone was effective in reducing, but not eliminating, the respiratory noises and dysphagia, with improved quality of life 763 days after initial presentation.

**Conclusion:**

This is a clinical description of an overlap syndrome of masticatory myositis and polymyositis, further complicated by lymphocytic thyroiditis. This case report highlights the importance of differentiating euthyroid sick syndrome and lymphocytic thyroiditis in dogs with chronic immune-mediated illness.

## Introduction

Canine inflammatory myopathies are characterized by the infiltration of inflammatory cells into striated muscle (1). Inflammatory myopathies may be generalized, affecting multiple muscle groups, or focal, limited to specific muscle groups, including the masticatory muscles in masticatory myositis or the extraocular muscles in extraocular myositis. Etiologies may be infectious, immune-mediated (e.g., polymyositis), or paraneoplastic. Clinical signs of generalized myositis frequently include generalized weakness, stiff gait, megaesophagus, and muscle atrophy. Creatine kinase (CK) activity is typically elevated; electromyography (EMG) usually shows abnormalities, with diffuse spontaneous activity and normal motor nerve conduction velocity; and an inflammatory pattern is usually found on skeletal muscle biopsies (2, 3). By contrast, masticatory myositis (MM) is a focal, immune-mediated inflammatory myopathy localized to the muscles of mastication (pterygoids, rostral digastricus, temporalis, and masseter muscles). Circulating antibodies against masticatory muscle type 2M fibers are typically found in the serum. Masticatory myositis may occur in dogs of any age and breed, but can be particularly severe in young dogs (4). Restricted jaw mobility can be found in both acute and chronic forms, whether the patient is awake or under anesthesia (1, 2, 5, 6). While the two most common immune-mediated myopathies in dogs are polymyositis and MM, only a few cases of overlap syndrome, characterized by inflammatory changes in multiple muscles, including the muscles of mastication, and a positive 2M antibody titer for MM, have been identified. One study involving 200 dogs with inflammatory myopathies identified three dogs with overlap syndrome (2). All three dogs had concurrent polymyositis and MM with autoantibodies against type 2M fibers, as in the dog in this case report.

Hypothyroidism is an endocrine disorder characterized by low serum thyroxine (T4) levels (7). Acquired primary hypothyroidism is the most common cause of hypothyroidism in the dog and is due to idiopathic thyroid gland atrophy or immune-mediated lymphocytic thyroiditis (LT). Diagnosis of immune-mediated LT is based on the presence of antithyroid antibodies in serum, with antithyroglobulin autoantibodies predominating; however, a study reported that elevated T3 autoantibodies may also be indicative of LT (8, 9). Accompanying changes may include low total T4, low free T4 (fT4), and high thyroid-stimulating hormone (TSH). Thyroid hormone levels can be affected by circadian and seasonal variations, medications, and non-thyroidal illness (10–16). Thyroid-stimulating hormone can also be normal in 13–38% of patients who have hypothyroidism (7, 8). Polymyositis-like syndrome is a reported sequela in people with untreated hypothyroidism, but it is rare or underreported in dogs. Therefore, measuring TSH is recommended in people presenting with muscle weakness or elevated serum CK activity (17). The typical changes in muscle associated with hypothyroidism include type 2 fiber atrophy, type 1 fiber predominance, and, on occasion, the presence of nemaline rod bodies (18). There have been reports of hypothyroid dogs exhibiting histopathological evidence of myopathy with or without associated clinical signs (19–21). The pathophysiologic mechanism is multifactorial and involves the downregulation of protein-encoding genes that regulate myocyte metabolism, plasticity, and neuromuscular transmission (18, 22).

Overlap syndrome encompasses multiple immune-mediated syndromes within the same patient and is well described in people. Here, we discuss a rare case of concurrent masticatory myositis, generalized immune-mediated polymyositis, and lymphocytic thyroiditis in a dog.

## Case description

A 4-year-old spayed female mixed breed dog was referred for evaluation due to an 8-to-10-month history of abnormal upper airway noises and dysphagia. These findings were first observed approximately 1 month after an exploratory celiotomy for a suspected gastrointestinal foreign body. There were no gross abnormalities noted intraoperatively, and a biopsy of a mesenteric lymph node was reported as reactive. The clinical signs were first described as waxing-and-waning nasal crusting, nasal pruritus, bilateral serous nasal discharge, and inspiratory stridor. Initially (day 1), the dog was prescribed 0.4 mg/kg/d prednisone and 1.9 mg/kg diphenhydramine PO q12h on a tapering schedule. On day 5, given the lack of improvement, prednisone was increased to 0.77 mg/kg/d and tapered over 2 weeks. This resulted in significant improvement in the nasal pruritus and discharge, but not the respiratory sounds. On approximately day 28, enrofloxacin was prescribed at 10 mg/kg PO q24h for 2 weeks; this reportedly resolved the respiratory sounds. The biochemistry profile revealed a mild elevation in alanine aminotransferase (ALT) activity of 175 U/L. Approximately 82 days from initial treatment, the dog was reported to have expiratory stertor and was prescribed clindamycin 13 mg/kg PO q12h for 2 weeks. The dog was rechecked on day 104, at which time some improvement was noted, and prednisone was refilled at an unknown dose. A biochemistry profile revealed ALT activity increased to 294 U/L. The dog remained largely unchanged, and referral to an internal medicine specialist was recommended.

On initial physical examination at the Veterinary Teaching Hospital 173 days after initial treatment, the dog was found to be over-conditioned with a body condition score of 7/9, mild bilateral rhinorrhea, intermittent inspiratory stridor, expiratory stertor with normal pulmonary sounds, multifocal erythematous epidermal collarettes with central hyperpigmentation on both flanks and the inguinal region, ventral alopecia, and generalized xerosis. On palpation, a firm enlargement of the ventral cervical region near the larynx was found along with severe symmetrical masticatory muscle atrophy, and trismus characterized by an inability to open the jaw more than approximately 3 cm. Additionally, the dog exhibited intermittent lateral strabismus in the left eye, ventrolateral strabismus in the right eye, and a mild right head tilt. An initial neurology examination revealed a fatigable palpebral reflex bilaterally, bilateral temporalis and masseter muscle atrophy, and trismus, which prevented the assessment of the gag reflex. Based on these findings, the neuroanatomic localization was the motor unit, with possible vestibular disease.

Hematology revealed a mild basophilia (0.090 × 10^3^/μL; RI 0.000–0.000), and serum biochemistry revealed increased ALT (202 U/L; RI 16–75) and CK (3,296 U/L; RI 32–193) activities, hypercholesterolemia (352 mg/dL; RI 129–332), and hypertriglyceridemia (201 mg/dL; RI 23–143). A free-catch urine specific gravity measured 1.030 through manual refractometry. A baseline serum cortisol measured 1.64 μg/dL (RI 1.00–7.13) and a serum total T4 via chemiluminescent immunometric assay measured <6.4 nmol/L (RI 16.0–37.7 nmol/L, detection limit of 6.4 nmol/L). A comprehensive thyroid panel consisting of total thyroxine, total triiodothyronine, free thyroxine by dialysis, T4 and T3 autoantibodies, thyroid-stimulating hormone, and thyroglobulin autoantibodies was submitted to a reference laboratory (Michigan State University Veterinary Diagnostic Laboratory, East Lansing, MI) to differentiate hypothyroidism from euthyroid sick syndrome. An ACTH stimulation test with a 1-h post-cortisol was 10.20 μg/dL (RI 4.10–19.90 μg/dL).

Three-view thoracic radiographs and fluoroscopy revealed intermittent primary wave dysmotility or primary wave depression. Although considered an unlikely differential, Leptospirosis is a cause of myopathy as reported in people. A bedside Leptospirosis IgM antibody test was negative. General anesthesia was performed for an airway examination, computed tomography (CT), electrodiagnostics, and muscle biopsies. The trismus was unchanged, and laryngeal examination revealed bilateral laryngeal paresis. Computed tomography of the head and neck was performed pre- and post-IV administration of iodinated contrast. The CT abnormalities included symmetrical thickening of the longus capitis, rectus capitis mentalis, and digastricus muscles, with heterogenous contrast enhancement of the masseter and temporalis muscles. In addition, symmetric tonsillitis (left 1.19 cm, right 0.65 cm) with moderate heterogenous contrast enhancement; mandibular, medial retropharyngeal, and prescapular lymphadenopathy; and asymmetry of the dorsocaudal nasopharynx were present.

An EMG examination was performed using a Cadwell Sierra Wave (version 11.0.116) with a concentric needle on the left plantar and palmar interosseous, cranial tibial, gastrocnemius, quadriceps femoris, semimembranosus and semitendinosus, lumbar epaxials, extensor carpi radialis, biceps brachii, triceps brachii, masseter, temporalis, and intrinsic tongue muscles. There was spontaneous activity recognized in all muscles tested, characterized by increased insertional activity, positive sharp waves, fibrillation potentials, and complex repetitive discharges. Spontaneous activity was most severe in the distal limb muscles and muscles of mastication. [(left pelvic limb: interosseous—2+ fibs/sharps, cranial tibial 3+ fibs/sharps with complex repetitive discharge, quadriceps 2+ fibs/sharps, lumbar epaxials normal), (left thoracic limb: interosseous 2+ fibs/sharps, extensor carpi radialis 3+ fibs/sharps with complex repetitive discharge, triceps 1+, biceps 2–3+), temporalis 4+ with complex repetitive discharge, masseter 4+ with complex repetitive discharge, tongue 4+ fibs/sharps]. Motor nerve conduction velocity was evaluated in the left common peroneal nerve. Compound muscle action potential amplitudes recorded at the level of the hip, stifle, and hock were 10.8 mV, 3.4 mV, and 3.8 mV, respectively (19.8 ± 1.4 mV) (23). Motor nerve conduction velocity measured from hip to hock was 51 m/s and 67 m/s from stifle to hock (60 ± 10 m/s) (24). Overall, this is suggestive of normal nerve function due to the normal amplitude and velocity of 67 m/s measured at one site. However, the amplitudes at the distal sites were low, with a normal waveform configuration, which could represent either axonal loss or an artifact of electrode positioning. Muscle biopsies were recommended.

**Figure 1:**
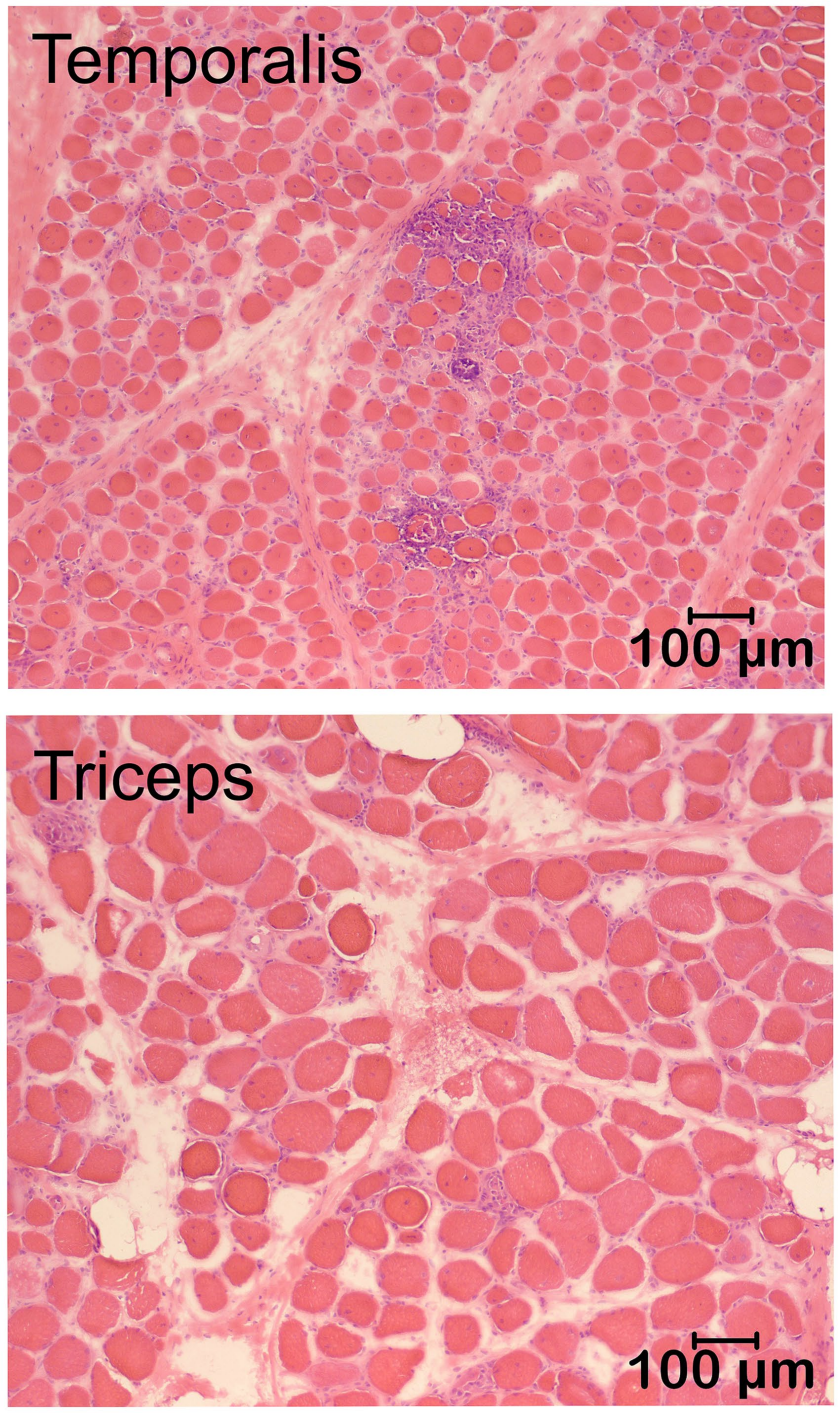
Frozen sections (8 μm) from the temporalis and triceps muscles show multifocal areas of mononuclear cell infiltration without obvious fiber loss and fibrosis. Hematoxylin and eosin stain, bar = 100 μm.

Unfixed, chilled and formalin-fixed biopsies were taken from the right cranial tibial, right triceps brachii, and right temporalis muscles and submitted to the Comparative Neuromuscular Laboratory, University of California, San Diego, by a courier service. The unfixed biopsies were evaluated in frozen sections using a standard panel of histochemical stains and reactions, including fiber typing, and the fixed biopsies were evaluated in routine paraffin sections (25). Multifocal areas of mixed mononuclear cell infiltrations were present in all three muscles (Figure 1), with infiltrating cells composed predominantly of lymphocytes and acid phosphatase/esterase reactive macrophages. Fiber type grouping was not observed. Intramuscular nerve branches were normal in appearance. Sporadic necrotic myofibers were undergoing phagocytosis within the temporalis and triceps muscles. Fibrosis, fiber loss, infectious organisms, or neoplastic cells were not obvious. A diagnosis of an inflammatory myopathy affecting both limb and masticatory muscles was made, with causes including infectious diseases, immune-mediated polymyositis, or a paraneoplastic syndrome.

Additional testing included determination of the serum 2M antibody titer by ELISA, which revealed a positive titer of 1:1000 (reference <1:100), consistent with a diagnosis of immune-mediated masticatory myositis. The serum acetylcholine receptor antibody titer was within the reference range (0.09 nmol/L, reference <0.6 nmoL/L). Serum antibody titers for *Neospora caninum* were negative. Paired convalescent serum antibody titers for *Toxoplasma gondii* revealed an initial IgM of 64 and IgG of 0, with both IgM and IgG measuring 0, 1 month later.

A dermatology consultation was performed, and skin cytology revealed no significant findings. Aerobic bacterial culture and susceptibility samples were submitted. The skin culture returned 2+ beta-lactamase-negative *Staphylococcus pseudintermedius* and 2+ *Staphylococcus aureus*, both of which were sensitive to a variety of antimicrobials. Superficial pyoderma was diagnosed. A clindamycin oral suspension was prescribed at 13 mg/kg PO q12h for 14 days.

The comprehensive thyroid panel showed the TT4 2 nmol/L (RI 9–52 nmol/L) measured via chemiluminescence immunoassay (CLIA), total T3 0.7 nmol/L (RI 0.8–2.1 nmol/L) measured by radioimmunoassay (RIA), fT4 by equilibrium dialysis 6 pmol/L (RI 6–42 pmol/L) measured by RIA, T4 autoantibody index 10% (RI 0–20%) measured by RIA, T3 autoantibody index 13% (RI 0–10%) measured by RIA, TSH 4.46 ng/mL (RI 0.00–0.58 ng/mL), measured by CLIA, thyroglobulin autoantibody index 105% (RI 0–35%) measured by ELISA, and specific binding thyroglobulin autoantibody index 114% (RI 0–25%) measured by ELISA. These findings were diagnostic of LT. The dog was discharged with topical 3% chlorhexidine digluconate and 0.5% ophytrium mousse, levothyroxine sodium 0.022 mg/kg PO, and a commercial therapeutic gastrointestinal diet. Prednisone 1 mg/kg PO q24h was prescribed following the negative skin culture and protozoal titers that returned 4 days later.

On day 208 post-initial treatment (33 days after prescribing prednisone and clindamycin), the recheck examination did not yield any changes. Further evaluation included Hepatozoonosis PCR, which was negative, and abdominal ultrasound, which showed mild jejunal muscularis thickening, bilateral adrenal gland atrophy likely secondary to prednisone therapy, mild renal diverticular mineralization, and gallbladder sludge. The prednisone dosage was increased to 1.8 mg/kg/day. Twelve days after increasing the prednisone dose, clinical improvement was seen, which included: improved mass of muscles of mastication, and resolution of right-sided vestibular signs. Prednisone was continued at 1.8 mg/kg/day and levothyroxine at 0.022 mg/kg/day. Recheck examination 41 days after starting immunosuppressive prednisone revealed increased ability to open the jaw, although the range of motion was still limited. Examination was otherwise unchanged. The prednisone was tapered every 14 days: 1.6 mg/kg/day, then 1.4 mg/kg/day, then 1.1 mg/kg/day, then 0.9 mg/kg/day, and then 0.7 mg/kg/day. Recheck examinations were performed and revealed continued improvement in the range of motion of the temporomandibular joint, although it was still not normal. The dog had also gained 1 kg in weight since immunosuppressive prednisone was started. Prednisone taper was continued at 0.35 mg/kg/day for 14 days, then 0.18 mg/kg/day for 14 days, then discontinued. Throughout treatment, the masseter and temporalis muscle atrophy did not improve except for the first recheck. Additionally, upper airway noises decreased in frequency but remained present, and dysphagia had also improved.

Unfortunately, 171 days after the initial start of the immunosuppressive steroids (24 days after being off prednisone), the dog had a relapse, described as increased muscle wasting and decreased ability to open the jaw. Creatine kinase activity was elevated (1,937 U/L; RI 32–193), and the serum 2M antibody titer was still positive at 1:1000. The dog restarted on 1.8 mg/kg/d of prednisone, and 2 weeks later, Azathioprine was initiated at 1.8 mg/kg daily for 1 week then decreased to 1.8 mg/kg every other day. Hematology was performed and consistent with a stress leukogram. Biochemistry profile revealed a mild hepatopathy with an ALT activity of 81 U/L (RI 16–75), ALKP activity of 196 U/L (RI 8–70), and GGT activity of 4 U/L (RI 1–5). Two weeks after azathioprine was initiated, hematology and biochemistry were performed. Hematology was again consistent with a stress leukogram; however, biochemistry was consistent with a worsening hepatopathy (ALT 995 U/L, ALKP 776 U/L, GGT 47 U/L). Azathioprine was therefore discontinued despite the improvement noted on physical and neurologic exams. After 2 weeks, the biochemistry profile improved (ALT 403 U/L, ALKP 457 U/L, GGT 46 U/L), and the dog was continued on 1.8 mg/kg/d of prednisone for 120 days, after which the prednisone taper was again attempted. The prednisone taper was performed every 4 weeks until after 350 days, when the dog was on 0.2 mg/kg/d of prednisone, at which dose the dog stayed indefinitely. At the last recheck examination, 763 days from the initial treatment by the veterinarian, the physical and neurologic examinations were like the examination prior to the relapse, with improved jaw range of motion and muscle mass from initial presentation, though the stertor was still present. Overall, the owner felt the dog had a good quality of life.

## Discussion

In this case report, an overlap syndrome of masticatory myositis with positive serum 2M antibodies, concurrent generalized inflammatory myopathy consistent with polymyositis, and lymphocytic thyroiditis is described in a dog. Overlap syndromes are defined as two or more immune-mediated diseases either simultaneously or sequentially (26). In humans, overlap syndrome or overlap myositis is common and is defined by myositis in addition to collagen disorders, systemic sclerosis, or systemic lupus erythematosus (27). Overlap syndrome in dogs was previously described in a retrospective study of inflammatory myopathy, in which 3 dogs were reported to have overlap syndrome (2). In that paper, all three dogs had concurrent generalized polymyositis and masticatory myositis with positive 2M antibodies, similar to the dog in this case report. The dog in this case report had evidence of three immune-mediated diseases, though, due to the chronic history at presentation, the order in which they developed is unknown. There are several reports of glandular tissue immune-mediated disease with concurrent LT, but the temporal relationship was unable to be determined (28–30). Of the three dogs with previously reported overlap syndrome characterized by generalized polymyositis and MM, one died, one was euthanized, and the third was lost to follow-up (2). This suggested a poorer prognosis for overlap syndrome, which was not found in this dog. Another case of overlap syndrome has also been reported in a dog with concurrent MM and myasthenia gravis, and it ultimately responded well to treatment. Additionally, a previous case of polymyositis, polyarthritis, and systemic lupus erythematosus was reported in a dog, which may more closely approximate the human form of overlap syndrome (31).

In this dog, CT was supportive of a generalized inflammatory myopathy, and MRI has previously been used to diagnose MM in dogs (32). We found symmetric enlargement of the musculature of the head and neck with subtle contrast enhancement. Although ultimately the diagnosis was reached in combination with diagnostic findings, CT with contrast or MRI may play a role in the diagnosis of myositis.

While this dog did have evidence of lymphocytic hypothyroidism, the muscle biopsies were not consistent with previous reports of pathologic changes in persistently hypothyroid dogs (18). Hypothyroid myopathy in dogs results in atrophy of type II (fast-twitch) myofibers, with a relative predominance of type I (slow-twitch) myofibers. In the previous study, six dogs were induced with hypothyroidism via IV irradiated iodine-131; none of these dogs initially had EMG abnormalities; however, five of six hypothyroid dogs developed progressive EMG abnormalities, including fibrillation potentials, positive sharp waves, and increased insertional activity, with all six exhibiting EMG abnormalities by the end of the study (18). Nemaline rods are also reported in persistently hypothyroid dogs (21). We did not see fiber-type grouping, relative change in the ratio of myofiber subtypes, nor nemaline rods in the current dog. This, combined with the inflammatory infiltrates, makes immune-mediated polymyositis a much more likely diagnosis than hypothyroid myopathy.

Other differential diagnoses considered were paraneoplastic syndrome, as described in a case report of primary cutaneous undifferentiated round cell tumor with concurrent polymyositis in a dog, and lymphoma-associated polymyositis in several dogs (33, 34). (Gianella, Neravanda) Infectious agents, such as leishmaniasis, sarcocystosis, Clostridioides, and Trichinellosis, were considered, but the lack of infectious organisms in the biopsies made these unlikely (35). Finally, other differential diagnoses include genetic or hereditary etiologies. Long-term follow-up may not be possible for the remainder of the dog’s life, which could result in delayed or missed diagnosis of other autoimmune polyglandular-like syndromes. Such examples include dogs with concurrent hypothyroidism and diabetes mellitus, or hypothyroidism and hypoadrenocorticism (29, 30, 36–39). Finally, other junctionopathies, such as myasthenia gravis, acute polyradiculoneuritis, and tick paralysis, were considered. Myasthenia gravis was considered very unlikely due to the negative acetylcholine receptor autoantibody titer and presence of spontaneous activity on EMG. Acute polyradiculoneuritis has EMG changes similar to those of inflammatory myopathies such as polymyositis, but nerve conduction velocities were normal, making it much less likely in this case. Tick paralysis was deemed unlikely given the lack of ticks on repeated physical exams and the chronicity of the clinical signs.

In conclusion, we report a novel case of a dog with masticatory myositis, diffuse polymyositis, and lymphocytic thyroiditis. A complete thyroid profile allowed rapid differentiation between lymphocytic thyroiditis and euthyroid sick syndrome. Supplementation with thyroxine, in addition to treatment for the inflammatory myopathies, likely improved the dog’s clinical signs and quality of life.

## Data Availability

The original contributions presented in the study are included in the article/supplementary material, further inquiries can be directed to the corresponding author.
